# Myocardial injury in a pediatric patient with diabetic ketoacidosis: A case report

**DOI:** 10.1097/MD.0000000000025702

**Published:** 2021-04-30

**Authors:** Houn Joe Shim, Byung Min Yoo, Seon Mi Jin, Min Jae Kang

**Affiliations:** Department of Pediatrics, Hallym University Sacred Heart Hospital, Hallym University College of Medicine, Anyang, Korea.

**Keywords:** child, diabetes complications, diabetic ketoacidosis, troponin I

## Abstract

**Rationale:**

Diabetic ketoacidosis (DKA) can cause several complications. Among them, cardiac complications are the most fatal and difficult to detect. Cardiac markers are prognostic factors for morbidity and mortality in adult patients with DKA. But, there have been very few discussed cases in pediatrics. We report a case of severe DKA in child with elevated cardiac enzymes and prolonged QT interval.

**Patient concerns:**

A 12-year-old girl admitted by nausea, vomiting, and lethargy for 1 day.

**Diagnoses:**

Her blood sugar level was initially undetectable by the capillary blood glucose meter, and blood gas analysis showed severe DKA with elevated cardiac enzymes and prolonged QT interval.

**Interventions:**

The patient was admitted to hospital and intensive intravenous fluid and regular insulin infusion were administered.

**Outcomes:**

After 5 days of supportive care, the patient was fully recovered, discharged, and followed up in an outpatient clinic.

**Lessons:**

Since the relationship between DKA and myocardial injury has not been clearly elucidated, pediatricians and emergency physicians should remain careful throughout the recovery time as it can lead to life-threatening conditions in various courses.

## 1 Introduction

Diabetic ketoacidosis (DKA) is considered the most urgent situation related to type 1 diabetes mellitus (DM). It usually occurs with new-onset diabetes or abortive management due to neglect or concurrent conditions.^[1]^ When treated inappropriately, DKA can cause several complications, including cerebral edema, vascular thrombosis, pulmonary edema, or various metabolic problems.^[1,2]^ Myocardial injury is a crucial condition related to mortality in patients with severe DKA, accounting for 28% of deaths in adult patients with DKA.^[3]^

The pathophysiological relevance between DKA and myocardial dysfunction is still obscure due to their complex mechanism, but it has been proposed that both conditions can trigger each other and mask significant clinical features, which leads to difficulty in diagnosis.^[4]^ Cardiac markers, including troponin I elevation, are prognostic factors for morbidity and mortality in adult patients with DKA, even without acute coronary syndrome.^[5–7]^ Nevertheless, there have been extremely few discussed cases in pediatrics. Here, we present a case of DKA with myocardial injury in a 12-year-old child to report and discuss the necessity of early and thorough cardiac evaluation in DKA patients in children and adolescents.

## 2 Case

A 12-years-old girl with previously diagnosed type 1 DM was admitted with complaints of nausea, vomiting, and lethargy for 1 day. She was first diagnosed with type 1 DM when she was 7-year-old (initial serum glucose was 508 mg/dL, serum C-peptide was 0.6 ng/mL, hemoglobin A1c was 12.7%, and anti-insulin antibody was positive). She did not have DKA at first diagnosis (pH 7.36 with bicarbonate level of 24.1 mEq/L and an anion gap of 7). One year after diagnosis, she skipped the follow-up clinic, and occasionally skipped insulin. She did not have any other medical issues or medications except for type 1 DM during this period. There was no family history of DM, metabolic syndrome, or cardiovascular disease. A review of systems revealed polyuria and polydipsia that started 2 days prior; nausea, vomiting, and abdominal pain developed after. In addition, she had a mild fever (reached above 38°C once) and pharyngeal injection, which implicates acute infection or inflammation.

At the time of arrival in the emergency room, she was lethargic but she had a clear mental status. Her blood pressure was 120/80 mm Hg, heart rate was 120 beats/min, respiratory rate was 22 breaths/min, body temperature was 37.3°C, and pulse oximetry was 100% on room air. Her height was 152 cm (25th–50th percentile), and her weight was 43.7 kg (25th–50th percentile) at admission. Later she was 45.2 kg at discharge time. Her blood sugar level was initially undetectable by the capillary blood glucose meter, and blood gas analysis showed a pH of 7.11 with a bicarbonate level of 7.1 mEq/L, and an anion gap of 24, therefore, all these findings were consistent with severe DKA (Table 1). Laboratory tests showed hyperglycemia (686 mg/dL), high hemoglobin A1c (8.2%), leukocytosis, (59,500/μL; 70.0% neutrophils, 16.0% lymphocytes), and a positive qualitative C-reactive protein (45 mg/L; upper limit of normal: 5 mg/L). Urinalysis showed glucosuria (3+), proteinuria (1+), and ketonuria (3+). The thyroid function test results were normal. Computed tomography (CT) of the brain was normal which was performed to screen for cerebral edema. Although serum creatine kinase level was in normal range, cardiac markers, such as creatine kinase myocardial band (4.4 ng/mL) and troponin I (104.0 pg/mL) were elevated (Table 2). But initial electrocardiogram (EKG) was normal with a corrected QT interval (QTc) interval of 464 ms.

**Table 1:** Initial and follow-up laboratory results.

	Reference	Unit	Initial	4 hour	10 hour	14 hour	Day 2	Day 3	Day 4
Venous pH	7.35–7.45	pH	7.117	7.211	7.332	7.342	7.348	7.385	7.423
Venous pCO_2_	35.0–45.0	mm Hg	21.9	28.7	28.4	32.1	34.1	31.7	34.2
Venous base excess	−3.0–3.0	mEq/L	−19.8	−16.4	−11.0	−8.4	−6.9	−6.1	−2.1
Venous bicarbonate	23.0–29.0	mEq/L	7.1	11.6	15.1	17.6	18.9	19.1	22.5
Serum glucose		mg/dL	686	275	105	149	197	276	134
BUN	5.0–18.0	mg/dL	32.0	23.0	ND	16.2	11.7	9.3	12.1
Creatine	0.4–0.6	mg/dL	1.6	1.2	ND	0.8	0.8	0.6	0.5
WBC	4000–10,000	unit/uL	59,500	ND	ND	33,800	ND	9500	ND
CRP	0.0–5.0	mg/L	45.0	54.5	ND	26.2	8.4	5.2	1.8

**Table 2:** Changes in cardiac markers.

	Reference	Unit	Initial	4 hour	Day 2	Day 3	Day 4	Day 6
CK	22–269	IU/L	140	127	102	67	47	49
CK-MB	0.0–3.4	ng/mL	4.4	5.3	ND	ND	ND	ND
Troponin I	0.0–15.6	pg/mL	104.0	172.0	158.8	142.3	158.8	29.1
NT-proBNP	0.0–186.0	pg/mL	ND	1290.0	319.4	59.99	78.82	<5

**Figure 1:**
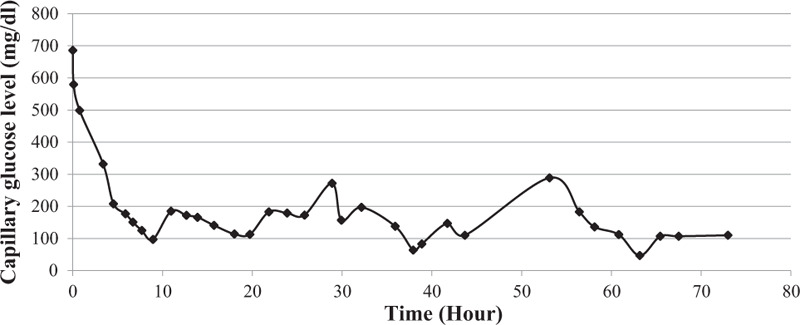
Changes in capillary blood glucose level was shown.

Intensive intravenous fluid and regular insulin infusion were administered, and blood gas analysis was performed every 2 hours to check and modulate metabolic changes. An empirical antibiotic (ampicillin/sulbactam 200 mg/kg/d) was also administered for pharyngeal injection and elevation of inflammatory markers. After 6 hours of treatment, her plasma glucose level decreased to 200 mg/dL (Fig. 1), and ketonuria disappeared. Her nausea, vomiting, and lethargy were relieved, but epigastric discomfort and mild abdominal pain persisted. Cardiac markers were significantly elevated compared to initial lab results (Troponin-I 172.0 pg/mL and N-terminal prohormone of brain natriuretic peptide 1290.0 pg/mL, Table 2), and a follow-up EKG showed borderline QT prolongation (QTc 477 ms) without ST elevations. An echocardiogram showed left ventricular diastolic abnormality (ejection fraction of 29%), indicating mild cardiac dysfunction, but the heart and coronary vessel structures and other cardiac functions were normal.

**Figure 2:**
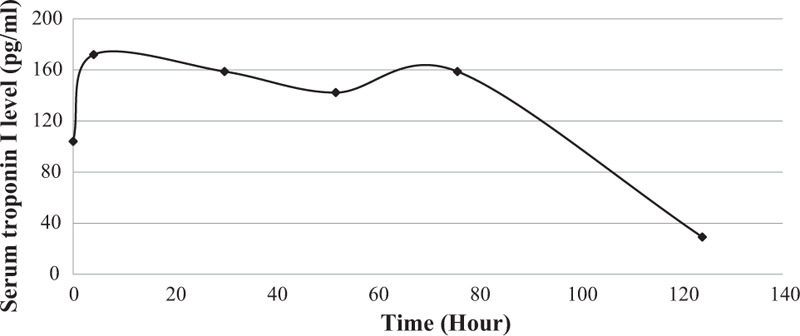
Changes in serum Troponin-I level was shown. It was normalized on hospital day 6.

On hospital day 2 (HD#2), acidosis and anion gap gradually improved (Table 1). Insulin infusion was discontinued after 39 hours of treatment, and subcutaneous insulin was restarted. Leukocytosis and elevated CRP levels were normalized at HD#2. Her vital signs were stable throughout in-hospital days. The respiratory viral tests were all negative. After 4 days of supportive care, cardiac markers were normalized at HD#6 (Troponin-I <10 pg/mL and N-terminal prohormone of brain natriuretic peptide < 5 pg/mL, Fig. 2), and serial EKG were normalized at HD#4 (QTc: 502 ms → 442 ms). Echocardiogram on HD#6 showed normalized left ventricular diastolic function (ejection fraction 33%), implying myocardial injury during recovery. She was discharged after completion of diabetes education, and tolerable glycemic control with intensive insulin therapy (Tresiba at night, Novorapid before every meal).

This study was approved by the institutional review boards of Hallym University Sacred Heart Hospital (IRB# 2020–06-011). The patient and her parents provided informed consent for publication.

## 3 Discussion

In adults with DM, acute cardiac decompensation is a common outcome due to early-onset atherosclerosis, while it is often less considered in children with DM. However, the relationship between DKA and myocardial dysfunction should not be overlooked. Although this case showed mild epigastric discomfort, other specific cardiac symptoms such as dyspnea and chest tightness were not observed during the illness. However, cardiac markers were elevated, serial EKG showed QT prolongation for the period of time, and echocardiogram showed subtle cardiac compromise in her left ventricle, indicating myocardial injury during DKA.

Several studies suggest that DKA leads to myocardial injury with multifactorial mechanisms.^[3]^ Electrolyte imbalance, including hyperkalemia, was accused of the main cause of arrhythmia, causing various degrees of AV block.^[8]^ Kuppermann et al found that QTc prolongation was observed in children with DKA without any electrolyte abnormalities and stated that ketotic conditions caused by DKA and insulin during the treatment contributed to this phenomenon.^[9]^ Batra et al reported a case of myocardial infarction related to the hyperosmolar state in children with DKA and suggested that accumulation of solute related to hyperosmolarity causes alteration of blood flow and subsequent impairment of erythrocyte flexibility, leading to cardiovascular disturbances.^[10]^ Recent studies also suggest that severe acidemia itself has an independent impact on myocardial stunning by irritating the activation of intercellular calcium and contractile proteins.^[11]^

Counter-regulatory hormones such as catecholamines are also suspected to be responsible for acute decompensation. These hormones increase the oxygen demand of the myocardium, resulting in myonecrosis with elevated troponin-I due to supply-demand mismatch.^[3,8]^ Japitana et al reported a case of stress myocardiopathy in a patient with DKA related to catecholamine excess from precipitating factors.^[2,12]^ Moller et al suggested that increased free fatty acid release due to ketoacidosis and counter-regulatory hormones leads to fatty acid incorporation and micelle formation in the myocardial plasma membrane, resulting in destabilization and rupture of the myocyte membrane.^[4]^ We cannot exactly reveal the direct cause of myocardial injury but based on initial blood gas results, we could assume that exposure to acidosis and elevated catecholamines to compensate this condition may majorly lead to myocardial injury in our case.

Although various aspects of DKA contribute to difficulty in predicting cardiac complications, previous reports showed that cardiac enzymes normalization took 5 to 10 days and myocardial injury was well-recovered after resolution of DKA as also seen in our case.^[10,12]^ However, its risk comes from the potency of developing life-threatening arrhythmia or heart failure. Early recognition and precise decisions prevent morbidity and mortality. Therefore, this case emphasizes the necessity of thorough cardiac monitoring including serial ECG and cardiac marker follow-up in children with DKA from onset to full recovery.

## Author contributions

**Conceptualization:** Min Jae Kang.

**Data curation:** Houn Joe Shim, Seon Mi Jin.

**Formal analysis:** Houn Joe Shim, Byung Min Yoo.

**Methodology:** Seon Mi Jin, Min Jae Kang.

**Project administration:** Min Jae Kang.

**Resources:** Byung Min Yoo, Seon Mi Jin.

**Supervision**: Kang MJ.

**Validation:** Seon Mi Jin, Min Jae Kang.

**Visualization:** Houn Joe Shim, Byung Min Yoo, Seon Mi Jin.

**Writing – original draft:** Houn Joe Shim, Byung Min Yoo, Seon Mi Jin.

**Writing – review & editing:** Min Jae Kang.
